# Class-specific school closures for seasonal influenza: Optimizing timing and duration to prevent disease spread and minimize educational losses

**DOI:** 10.1371/journal.pone.0317017

**Published:** 2025-01-23

**Authors:** Yukiko Masumoto, Hiromi Kawasaki, Ryota Matsuyama, Miwako Tsunematsu, Masayuki Kakehashi

**Affiliations:** 1 Department of School and Public Health Nursing, Graduate School of Biomedical and Health Sciences, Hiroshima University, Hiroshima, Japan; 2 Faculty of Health and Welfare, Department of Welfare, Seinan Jo Gakuin University, Fukuoka, Japan; 3 Department of Veterinary Medicine, School of Veterinary Medicine, Rakuno Gakuen University, Ebetsu City, Hokkaido, Japan; 4 Department of Health Informatics, Graduate School of Biomedical and Health Sciences, Hiroshima University, Hiroshima, Japan; The University of HongKong, HONG KONG

## Abstract

School closures are a safe and important strategy for preventing infectious diseases in schools. However, the effects of school closures have not been fully demonstrated, and prolonged school closures have a negative impact on students and communities. This study evaluated class-specific school closure strategies to prevent the spread of seasonal influenza and determine the optimal timing and duration. We constructed a new model to describe the incidence of influenza in each class based on a stochastic susceptible-exposed-infected-removed model. We collected data on the number of infected absentees and class-specific school closures due to influenza from four high schools and the number of infected cases from the community in a Japanese city over three seasons (2016–2017, 2017–2018, and 2018–2019). The parameters included in the model were estimated using epidemic data. We evaluated the effects of class-specific school closures by measuring the reduced cumulative incidence of class closures per day. The greatest reduction in the cumulative absences per day was observed in the four-day class closure. When class-specific school closures lasted for four days, the reduction in the cumulative number of infections per class closure day was greater when the closure was timed earlier. The highest reduction in the number of class closures per person-day occurred when the threshold was around 5.0%. Large variations in the reduction of cumulative incidence were noted owing to stochastic factors. Reactive, class-specific school closures for seasonal influenza were most efficient when the percentage of newly infected students exceeded around 5.0%, with a closure duration of four days. The optimal strategy of class-specific school closure provides good long-term performance but may be affected by random variations.

## Introduction

In many schools, children frequently spend time together, which increases the risk of infection [1,2]. Children have immature immune systems and a high rate of human contact at school [3–5], resulting in high infection rates [6,7]. Therefore, outbreaks of infectious diseases in schools can spread rapidly and negatively impact families [6,8] and communities [9–11]. Controlling infectious diseases in schools can prevent their spread [12] and is necessary to protect children [13].

School closure is a nonpharmacological intervention [14–16]. The possible advantages of such an intervention include no direct economic cost, no side effects, and the ability to be implemented immediately once the decision is made by the school. School closures are a countermeasure to the spread of infectious diseases in schools, such as during the 2009 H1N1 Influenza Pandemic [16,17] and COVID-19 (SARS-CoV-2 virus) pandemics [18,19]. However, school closures can have harmful effects. Healthy children must remain absent from school. Prolonged school closures disrupt educational activities, impact student achievement [20,21], and negatively affect students’ mental health [22,23]. Additionally, they create challenges related to the need for childcare [24] and parents’ absenteeism from work [25]. Parental absenteeism disrupts social functioning and leads to increased social and economic costs [26,27]. Therefore, decisions regarding school closures must be made with careful consideration to minimize these adverse effects.

In Japan, reactive class closures occur during seasonal influenza epidemics. Schools can temporarily close all or a part of a school if needed to prevent infectious diseases (School Health and Safety Act) [28]; however, there are no standards for grades, classes, timing, or duration. In fact, timing and closure durations have not been sufficiently evaluated or standardized [29]. Therefore, the principal of each school decides which units in the school will be closed, and when, in consultation with the *Yogo* teachers (whose role is to support the growth and development of students through health education and health services in Japanese schools and analyze students’ health information on an ongoing basis), teachers, and school physicians [30]. Teachers should consider both the prevention of infection and the continuance of students’ education. Understanding the optimal timing (when a percentage of the class is infected) and duration (for how many days) of closures will enable school administrators and teachers to choose the best class-specific school closures (hereafter, “class closures”).

Several studies have evaluated reactive class closures for seasonal influenza [2,14,31–33]. The timing and duration of class closures continue to be debated [29,31], and the World Health Organization has indicated that more research is needed [16]. Mathematical modeling is a powerful tool for examining and quantifying the effects of intervention strategies [34,35] and is also used to examine the impact of class closures during pandemics [36,37]. Regarding the effects of class closures during the 2009 H1N1 Influenza Pandemic, Kawano and Kakehashi estimated the number of infected students in an entire city using a mathematical model [38]. They reported that class closures lasting four or more days were effective and indicated that further class-based studies are needed. In previous studies on influenza-like illnesses, targeted class closure strategies were as effective as whole-school closures at a much lower cost [39]. Endo et al. [2] used a mathematical model to estimate the reproduction number, revealing that more than half of the infections were attributable to within-class transmission. Class closure strategies are considered effective, and understanding the optimal timing and duration of such closures could aid in decision-making about implementing class closures.

This study evaluated class closure strategies for seasonal influenza to determine the optimal timing and duration. For our study, three years of four high school epidemic data and community epidemics were obtained from Japan. We constructed a new mathematical model for class closure and quantitatively evaluated its effects on seasonal influenza cases. In addition, we examined the most efficient timing and closure duration.

## Materials and methods

### Data collection

We analyzed data on the incidence of seasonal influenza in schools and the community over three seasons: 2016–2017, 2017–2018, and 2018–2019. School data collection was based on high school records, while data for community residents were obtained from a website. Data were obtained from Iwakuni City, which had a population of approximately 130,000 residents and 3,800 individuals aged 16 to 18 years in 2018.

### High school epidemic data

This study focused on four high schools in Iwakuni City. These schools were in the center of the city and within 2–6 km of each other. In these high schools, students usually attend classes and have lunch with the same class members. Classrooms are located on the same floor for each grade level.

Data were collected at each high school and including the number of students, the number of absences due to influenza, and whether classes were closed (May 13–31, 2019). The number of students was collected by class, and the number of classes was collected by grade level. Data were collected over a three-year period from 193 classes, which had 6,799 students across four schools (27 classes, 1,077 students; 36 classes, 1,304 students; 65 classes, 2,419 students; and 65 classes, 1,999 students, respectively). Third grade students in all schools were excluded from the analysis because they attended fewer days of school after January, which is before graduation, and there were no outbreaks in these classes. Therefore, 4,507 students in 125 classes in the first and second grade were included in the analysis. The average class size was 36.0 (SD ± 5.30) in 2016–2017, 36.5 (SD ± 4.46) in 2017–2018, and 36.0 (SD ± 5.88) in 2018–2019. Influenza A was reported in all season-years, while influenza B was reported only in 2017–2018.

Data on student absences due to influenza were collected through a case list of students who were absent because of influenza. The case list included the grade level, class, influenza virus type (A, B, or unspecified), and start and end dates of the closure. Students’ names and sex were removed from the list for anonymity. The list was based on a document that students submitted to the school. This documentation is intended to allow students to receive credit for the classes they missed. On this form, the examining physician entered a diagnosis of influenza and the duration of students’ absenteeism. In Japan, absenteeism due to seasonal influenza is defined as lasting at least five days after illness onset, including two days after the onset of a fever. Data on class closures owing to influenza were collected, including the affected classes and the start and end dates of the closures.

### Community epidemic data

Data on the number of influenza cases in Iwakuni City were collected from the Yamaguchi Prefectural Infectious Disease Information Center website [40] (accessed August 20, 2020). The number of cases and the reporting date were recorded. Since data were reported only on a weekly basis, the number of cases per week was divided by seven to estimate the incidence rate per day for each week. The influenza virus type was not noted in the records.

All data were completely anonymized prior to access; that is, none of the data included personal information such as name, sex, or address. The authors did not have access to any information that could identify individual participants during or after the data collection process. This study was approved by the Kibi International University Ethics Committee (no. 18–33; October 17, 2018). Following ethical approval, written and verbal consent was obtained from the high school headmaster. Data collection took place only after obtaining consent. Upon changing affiliated research institutions, the new institution conducted an ethical review and granted approval (the Hiroshima University Epidemiological Research Ethics Committee; no. E-2598; September 15, 2021).

### Model

To estimate influenza transmissibility, we employed a stochastic Susceptible-Exposed-Infectious-Recovered model as an extension of the Kermack–McKendrick model [41]. Our model describes the daily probability of infection among students, accounting for the force of infection at various levels: classroom, grade, school, and community. This extends the models from our previous studies [38,42].

**Fig 1:**
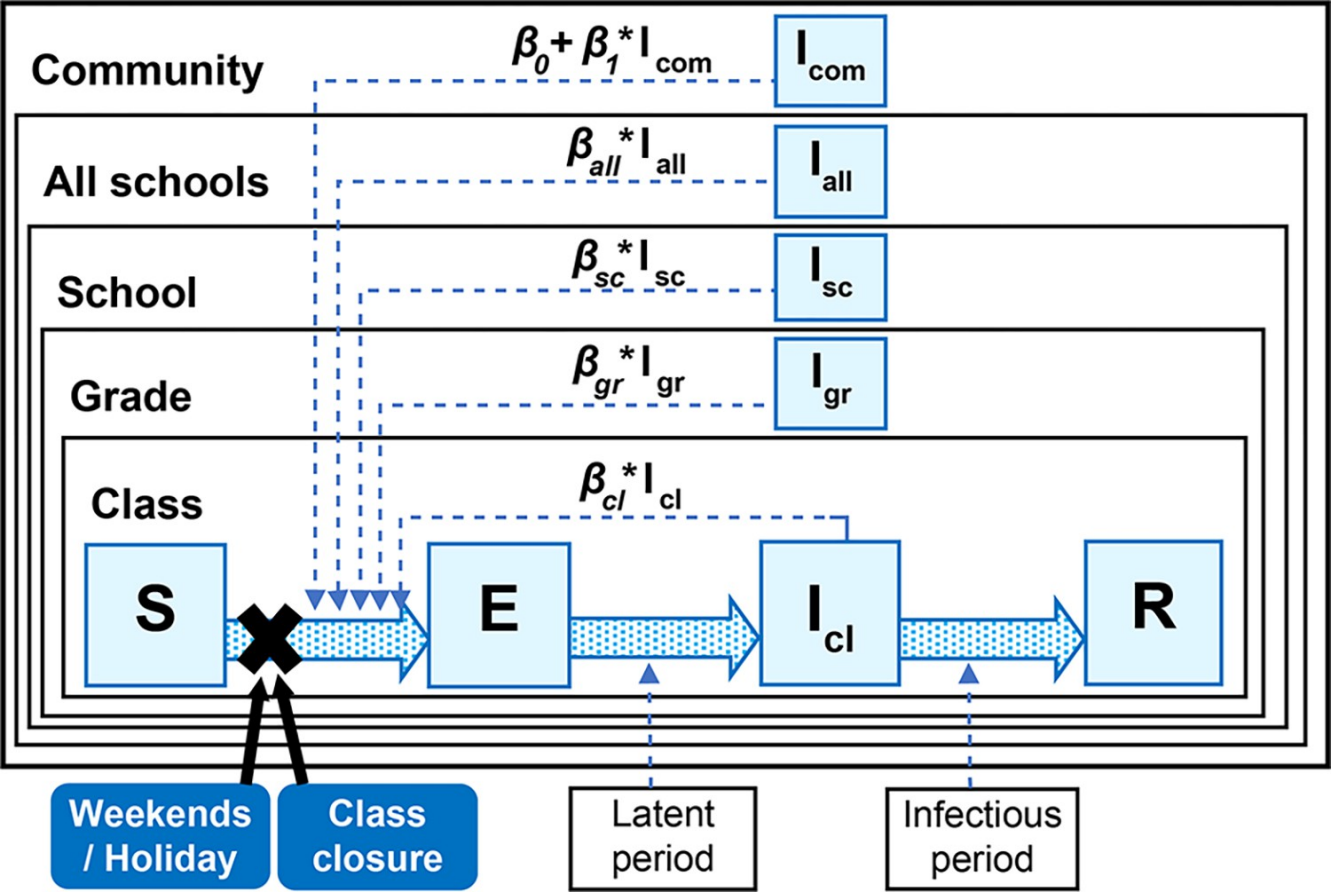
**Model structure.** *β*_*cl*_, *β*_*gr*_, *β*_*sc*_, *β*_*all*_, *β*_*0*_, and *β*_*1*_ are the transmission rates of infectious students within the same class, grade, and school, between schools, and from the community (intercept and coefficient), respectively (Fig 1).

Fig 1 outlines the structure of the model, based on a hierarchical population structure: community, schools, grades, and classrooms. The model includes six different transmission coefficients (*βs*): within class (*β*_*cl*_), within grade (*β*_*gr*_), within school (*β*_*sc*_), between schools (*β*_*all*_), and two different community-based coefficients (*β*_*0*_ and *β*_*1*_). The parameter *β*_*0*_ represents the intercept of community infections. Class (*cl*) is nested within grade (*gr*), and grade (*gr*) is nested within school (*sc*). Additionally, temporal variation in the force of infection is represented by B-spline basis functions *BSp1(t)*, *BSp2(t)*,*…*, *BSp5(t)* with parameters *s1–s5*.


BSp(t)=s1BSp1(t)+s2BSp2(t)+BSp3(t)+s4BSp(t)+s5BSp5(t) (1)

We set *s3* = 1 to avoid indeterminate parameters. Consequently, the model includes 10 transmission coefficients (Fig 1).

The pre-infectious and infectious periods for influenza were assumed to be one day and two days, respectively, based on the reported characteristics of the disease [43–45]. We also considered the effect of weekends/holidays in mitigating infection risk [46–48]. Weekends/holidays are expressed with an indicator variable (h), where h = 1 indicates weekends/holidays and h = 0 otherwise. Our main focus, the effect of class closures, was modeled by setting *β*_*cl*_, *β*_*gr*_, and *β*_*sc*_ to zero for students in closed classes [49–51].

Based on the model and the specified parameters, we calculated the probability of infection (*p*_*cl*_(*t*)) for susceptible students in a particular class at time t:

$$p_{cl}(t)=1-e^{-\lambda_{cl}(t)}$$ (2)

where *λcl* (t) denotes the force of infection on susceptible students.

It is vital to distinguish between incidence, prevalence, and the number of reported infections. Incidence refers to newly infected cases, typically reported after a delay. Prevalence refers to the number of infected individuals on any given day, including those already counted as infected. We derived the force of infection (*λ*(*t*)) using the prevalence ($\hat{I}(t)$) calculated from the incidence (*I*(*t*)). Let *I*_*rep*_(*t*) denote the number of reported cases of students with influenza on a given day, and the incidence *I*_*inc*_(*t*) is represented by *I*_*rep*_(*t*+1) if we assume that reporting is just one day after infection actually took place. Assuming the infection period for seasonal influenza is two days [43–45], the prevalence *I*_*prev*_(*t*) can be calculated as *I*_*prev*_(*t*) = *I*_*inc*_(*t*−2)+*I*_*inc*_(*t*−1), which we denote as $\hat{I}(t)$.

According to our assumptions, the force of infection, λ_*cl*_(t), was defined as follows:

$$\lambda_{sc,gr,cl}(t)\equiv \lambda (sc,gr,cl,t,\beta_{cl},\beta_{gr},\beta_{sc},\beta_{all},\beta_{0,}\beta_{1})$$

$$=Bsp(s_{1},s_{2},s_{4},s_{5},t)\{\left(1-h)(\beta_{cl}{\hat{I}}_{cl}(sc,gr,cl,t-1)+\beta_{gr}{\hat{I}}_{gr}(sc,gr,t-1){+\beta }_{sc}{\hat{I}}_{sc}(sc,t-1)+\beta_{all}{\hat{I}}_{all}(t-1))+\beta_{0}+\beta_{1}{\hat{I}}_{com}(t-1\right)\},$$ (3)

where *I*_*cl*_, *I*_*gr*_, *I*_*sc*_, *I*_*all*_, and *I*_*com*_ denote the number of infectious students within a class, grade, school, between schools, and in the community, respectively. On weekends/holidays (i.e., h = 1) or during class closures (i.e., *β*_cl_ = *β*_gr_ = *β*_sc_ = 0), *λ*_cl,gr,sc_(*t*) is equivalent to $\beta_{0}+\beta_{1}{\hat{I}}_{com}(t)$. The terms ${\hat{I}}_{gr}(sc,gr,t-1),{\hat{I}}_{sc}(sc,t-1),{\hat{I}}_{all}(t-1)$, and ${\hat{I}}_{com}(t-1)$ are the prevalence numbers for a grade, school, all schools, and the community:

$${\hat{I}}_{gr}(sc,gr,t)=\sum_{cl\in S(sc,gr)}{\hat{I}}_{cl}(sc,gr,cl,t),$$ (4)

$${\hat{I}}_{sc}(sc,t)=\sum_{gr\in S(sc)}{\hat{I}}_{gr}(sc,gr,t),$$ (5)

and

$${\hat{I}}_{all}(t)=\sum_{sc\in S(sc)}{\hat{I}}_{sc}(sc,t).$$ (6)

Here, *S* (*sc*,*gr*) and *S*(*sc*) represent the sets of all classes within a grade and all grades within a school, respectively. The number of community infections, ${\hat{I}}_{com}$, was obtained from weekly reports from Yamaguchi Prefectural Surveillance Center [40]. Although not the absolute number of community infections, the reported cases are assumed to be proportional to the total number of infected individuals in Iwakuni City, with *β*_1_ absorbing the scaling effect for Iwakuni City’s population.

### Parameter estimation

We assumed that infection events follow a binomial process modulated by the probability of infection, *p*_*cl*_(*t*). The binomial process is commonly used to simply model the probability of occurrences. While there are more complex models that account for host heterogeneity, we used this simpler approach because we had no further information about host heterogeneity. The likelihood function for the observed dynamics of infected students in each year was described as follows, using the probability of infection *p*_*cl*_(*t*):

$$=\prod_{t}\prod_{Cl}\left(\begin{array}{c} S_{cl}(t)\\ I_{cl}(t) \end{array}\right){p_{cl}(t)}^{I_{cl}(t)}{\left(1-p_{cl}(t)\right)}^{S_{cl}(t)}$$ (7)

where *S*_*cl*_(*t*) is the number of susceptible students at calendar time *t* in class *cl*, and *I*_*cl*_(*t*) represents the number of newly infected students (i.e., incidence) at time *t* in class *cl*. The parameters were estimated using maximum likelihood estimation by taking the logarithm of the likelihood function.

The probability of infection is related to the force of infection, *λ*. The force of infection is the sum of several types of infectious individuals (i.e., prevalence numbers) weighted by transmission parameter *β* of each type. In our model, the groups include individuals in the same class, grade, school, different schools, and community members. The transition parameters *β*_*cl*_,*β*_*gr*_,*β*_*sc*_, and *β*_*all*_, correspond to these groups, respectively. The weighted sum of infectious individuals is *β*_*cl*_*I*_*cl*_(*t*)+*β*_*gr*_*I*_*gr*_(*t*)+*β*_*sc*_*I*_*sc*_(*t*)+*β*_*all*_*I*_*all*_(*t*), where *I*_*cl*_(*t*),*I*_*gr*_(*t*),*I*_*sc*_(*t*), or *I*_*all*_(*t*) represent the number of infectious individuals in each group, respectively. The influence of the community is represented by *β*_0_+*β*_1_*I*_*com*_(*t*) if we write the number of community members as *I*_*com*_(*t*). We also consider temporal variation in the strength of infection, denoted by *s*(*t*), which is represented by $s(t)=S_{1}{BSp}_{1}(t){+S}_{2}{BSp}_{2}(t)+{S_{3}{BSp}_{3}(t)+S}_{4}{BSp}_{4}(t)+S_{5}{BSp}_{5}(t)$, with B-spline basis functions, ${BSp}_{1}(t),{BSp}_{2}(t),{BSp}_{3}(t),{BSp}_{4}(t),and\;{BSp}_{5}(t)$. We set *S*_3_ = 1 to eliminate ambiguity. The force of infection is as follows:

$$\lambda (t)\equiv s(t)\left(\beta_{cl}I_{cl}(t)+\beta_{gr}I_{gr}(t)+\beta_{sc}I_{sc}(t)+\beta_{all}I_{all}(t)+\beta_{0}+\beta_{1}I_{com}(t)\right)$$ (8)

We then have the following:

$$p_{cl}(t)=1-exp(-\lambda (t))$$ (9)

The probability of not getting infected is *e*^−*λ*^. The probability of getting infected is 1−*e*^−*λ*^ and can be approximated with *λ*(≅1−*e*^−*λ*^) when λ is small. Under this approximation, the force of infection (λ) multiplied by the number of susceptibles implies the incidence (i.e., the number of newly infected individuals). Incidence in the same class, grade, school, and in different schools is calculated as follows: Σ_*t*_*S*_*cl*_(*t*)*s*(*t*)*β*_*cl*_*I*_*cl*_(*t*), Σ_*t*_*S*_*gr*_(*t*)*s*(*t*)*β*_*gr*_*I*_*gr*_(*t*), Σ_*t*_*S*_*sc*_(*t*)*s*(*t*)*β*_*sc*_*I*_*sc*_(*t*), and Σ_*t*_*S*_*all*_(*t*)*s*(*t*)*β*_*all*_*I*_*all*_(*t*), respectively.

In summary, the likelihood function is as follows:

$$L(\beta_{cl},\beta_{gr},\beta_{sc},\beta_{all},\beta_{0,}\beta_{1,}S_{1},S_{2},S_{4},S_{5})$$

$$=\prod_{t}\prod_{Cl}\left(\begin{array}{c} S_{cl}(t)\\ I_{cl}(t) \end{array}\right){p_{cl}(t)}^{I_{cl}(t)}{\left(1-p_{cl}(t)\right)}^{S_{cl}(t)}$$ (10)

### Monte Carlo simulations for quantifying class closure effects

Using the estimated transmission coefficients, we simulated the dynamics of influenza under the Monte Carlo framework. According to the assumptions in our modeling, the number of susceptibles, exposed individuals, infectives (here prevalence number calculated based on incidence), and recoveries in a certain class (*cl)*, denoted as *S*_*cl*_(t), *E*_*cl*_(t), ${\hat{I}}_{cl}$ and *R*_*cl*_(t), can be written as follows:

$$S_{cl}(t+1)∼BinomialDistribution(S_{cl}(t),exp(-\lambda (t))),$$ (11)

$$E_{cl}(t+1)=S_{cl}(t)-S_{cl}(t+1),$$ (12)

$${\hat{I}}_{cl}(t+1)=E_{cl}(t)+E_{cl}(t-1),$$ (13)

and

$$R_{cl}(t+1)=R_{cl}(t){+E}_{cl}(t-2),$$ (14)

where the function *λ*(*t*) is the force of infection already described above.

We also considered various strategies for class closures, including timing and duration. We assumed that closures began when the number of infected students reached a certain percentage of the class population. Five thresholds (2.0%, 4.0%, 5.0%, 6.0%, and 8.0%) were considered as the criteria for the onset of closures, and five different durations were used (0, 2, 3, 4, and 5 days), creating 25 distinct patterns (5 thresholds × 5 durations). Each setting was simulated over three influenza seasons, generating 75 simulation outcomes. To maintain realistic population structure settings, we employed the same class, grade, school, and community structure as in the collected data. We ran 10 000 simulations and calculated the cumulative incidence and its variance for each setting.

### Epidemiological outcomes

Mathematica 14 software was used for the simulations. For each condition, we evaluated i) the total number of infected students (average number of final cases in the population), ii) the total number of classes and students absent due to class closures, and iii) the reduced number of cumulative absentees per day of class closure. The reduced cumulative incidence per person-day of class closure was used to evaluate several combinations of timing (starting criterion) and duration of class closures.

## Results

### The number of infected individuals in high schools and the community

The community epidemic followed a bimodal distribution, with one peak occurring during the holiday season and the other peaking in early February. In contrast, the high school epidemic peaked earlier than the regional epidemic in the 2016–2017 season, whereas no significant peaks were observed in the other two seasons. In the 2016–2017, 2017–2018, and 2018–2019 seasons, the reported cases were 264 (cumulative incidence: 17.1%; type A cases: 264), 285 (cumulative incidence: 19.0%; type A cases: 76; type B cases: 209), and 184 (cumulative incidence: 12.5%; type A cases; 184), respectively. The average seasonal infection incidence in the four schools ranged from 8.9 to 12.0% (0.0–56.3% per classroom). The time series of new infections in high schools and the community over these three years is provided in S1 Fig.

### The effect of class closure

**Fig 2:**
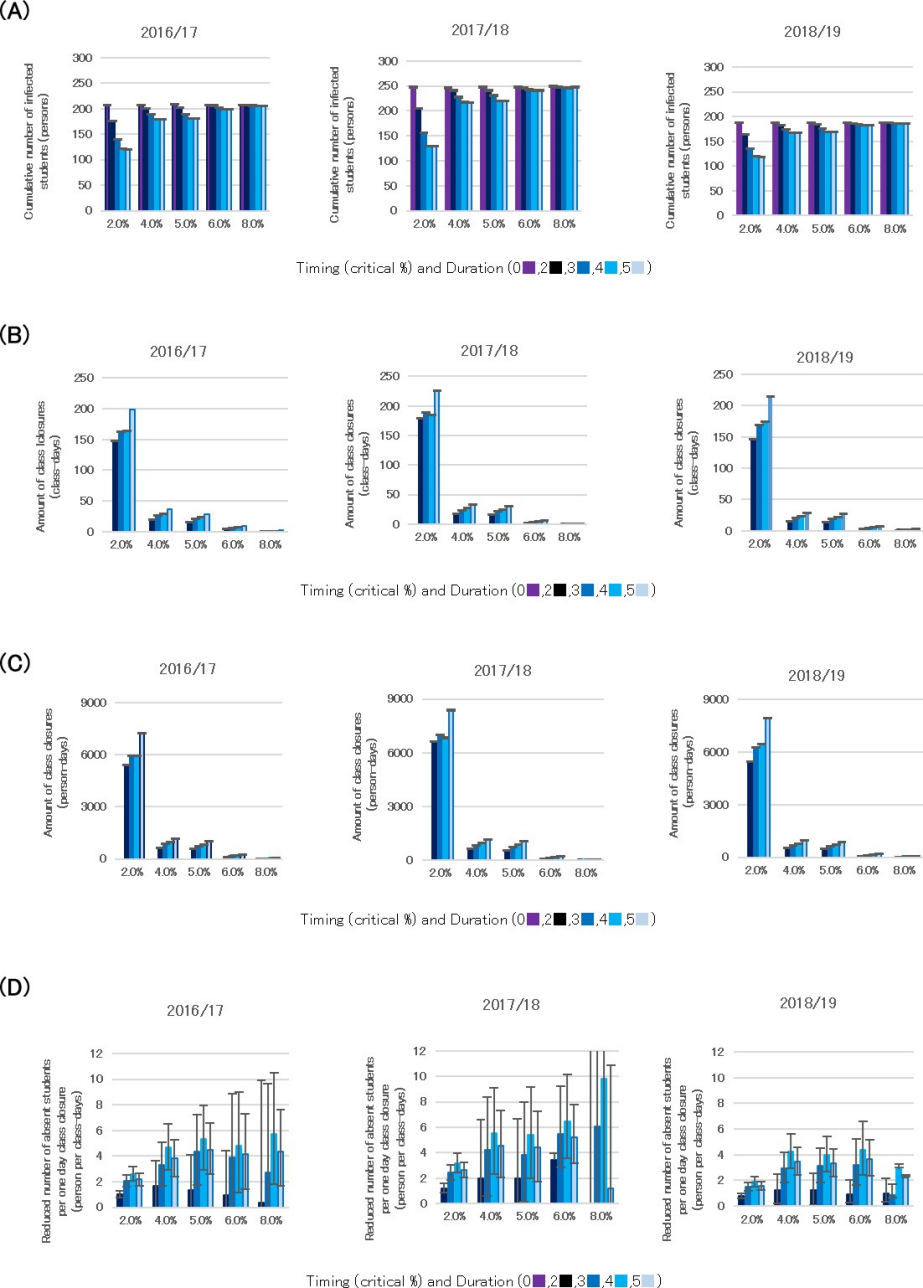
Simulation results for 25 class closure patterns. A: Cumulative number of infected students. B: Total class closures, calculated as class-days. C: Total class closures, calculated as person-days. D: Reduction in absent students per day of class closure. Simulations were performed with five different start times for class closures, based on the percentage of infected students (i.e., 2.0%, 4.0%, 5.0%, 6.0%, and 8.0%) and five class closure durations (0, 2, 3, 4, and 5 days). A duration of 0 days indicates the strategy of no class closures. Fig 2A shows the average cumulative number of infected students. Fig 2B shows the number of class closure in class-days, calculated by multiplying the number of closed classes by the closure duration for each scenario. Fig 2C shows the number of class closures in person-days, calculated by multiplying the number of infected students by closure duration for each scenario. Fig 2D shows the reduced number of cumulative absent students per day of class closure. This was calculated as follows: ([average number of infected students with no class closure − average number of infected students in each condition] × 5 days) / average number of class-days in each condition. Infected students were assumed to remain absent for five days (Fig 2).

Model parameters (*β*) were estimated using maximum likelihood estimation (S1 Table). The reproduction number was calculated from the estimated *β*. The reproduction number R(t) for high school students was 1.38, 1.27, and 1.11 in the 2016–2017, 2017–2018, and 2018–2019 seasons, respectively. The R(t) within classes (*βcl*) was 0.64, 0.29, and 0.53, respectively, and the infections were observed both within classes and between schools (S2 Table). The cumulative number of infections from the simulations was 206.9 for 2016–2017 (data: 264), 187.5 for 2017–2018 (data: 184), and 247.6 for 2018–2019 (data: 285). Fig 2 shows the simulation results for class closures over these three years using the estimated parameters. In this context, absence includes the total number of infected students and students who were not infected but remained absent because of class closures. Fig 2A shows the cumulative number of infected students. The greatest reduction in infections occurred when class closures were initiated at a 2.0% infection rate, and the per-day number of infected students decreased as the closure duration increased from 0 to 5 days. There was large variability in the probabilistic reduction of cumulative incidence. Fig 2B shows the number of class closures calculated as class-days (i.e., cumulative duration of class closures), which was calculated by multiplying the number of closed classes by the closure duration for each setting. The highest average number of class-days was observed when class closures started at 2.0%, and the number of class-days increased as the duration increased. Fig 2C shows the number of class closures calculated as person-days. Person-days (i.e., the cumulative number of student absences caused by class closures) were calculated by multiplying the number of infected students by class closure duration for each setting. Person-days were also highest when class closures started at 2.0%, and the number of absences per day increased as the duration increased. Fig 2D shows the reduction in the number of absent students per day of class closure, calculated as follows:

([average number of infected students with no class closure − average number of infected students in each condition] × 5 days) / average total number of class-days in each condition.

Infected students were absent for five days, in accordance with legal standards [52]. A four-day class closure resulted in the largest reduction in absences across all closure timing thresholds (Fig 2).

Table 1 shows the effects of four-day class closures, highlighting the reduction in the cumulative number of infected students, the number of class-days per one day of closure, and the reduction in absent students per one day of closure. The results show that the cumulative number of infected students and the number of class-days per day of closure decreased as class closures were implemented earlier. The highest reduction in absent students per day of closure occurred when closures were initiated at approximately 5.0% infection rates. These results indicate that the most efficient class closures, which minimize both the number of infected students and total absentees (including non-infected students), occur when the percentage of infected students exceeds around 5.0% and the closure duration is four days (Table 1).

**Table 1:** Effects of four-day class closures.

	Timing	2016–2017	2017–2018	2018–2019
**Reduced number of cumulative infected students (person-days)** **[= Δ]**	2.0%	85.041	117.72	67.41
	4.0%	27.819	29.28	20.35
	5.0%	26.54	27.46	18.07
	6.0%	7.68	6.53	4.81
	8.0%	1.37	3.38	1.56
**Number of class-days per one day of class closure (class-days)** **[= class-day / closure duration]**	2.0%	40.86	46.11	43.75
	4.0%	7.42	6.87	5.96
	5.0%	5.98	6.40	5.59
	6.0%	2.07	1.32	1.47
	8.0%	0.43	0.26	0.75
**Reduced number of absent students per one day of class closure (persons per class-day)** **[= 5Δ / class-day]**^**a**^	2.0%	2.61	3.18	1.92
	4.0%	4.66	5.52	4.27
	5.0%	5.32	5.39	4.01
	6.0%	4.82	6.47	4.35
	8.0%	5.73	9.83	2.90

^a^The standard duration of absence due to seasonal influenza was five days [52].

## Discussion

This study examines the effects of class closures on seasonal influenza. It is important to incorporate realistic transition structure and distribution in the model [53,54]. We incorporated hierarchical structure: i.e., classes, grades, schools, whole students and community, and assigned different transmission rates to each of the levels. The transitions at the whole student level and community level could represent the contacts due to social behavior outside schools. We then estimated parameters based on actual epidemic data from high schools and the community, including latent and infectious periods.

The estimated parameter (*β*) was the largest within classes, followed by within grades in all seasons, which aligns with previous studies showing high infection rates in classrooms [2]. The estimated R, ranging from 1.11 to 1.38, aligns with that of a previous study, which reported an R of 1.28 [42]. In addition, the cumulative number of infections in the simulation, ranging from 187.5 to 247.6, aligns with the observed data of 184 to 285. These results suggest that the parameter estimation was appropriate.

During the peak of the epidemic, R(t) was highest within classes in the 2016–2017 and 2018–2019 seasons. In contrast, in the 2017–2018 season, the R(t) between schools was high, suggesting that there were many opportunities for infection outside of school.

Simulation results, using the estimated parameters, indicated that the most efficient timing and duration to minimize infections and absences due to class closures were when the number of infected students exceeded around 5.0% and the closure duration was four days.

A previous study suggested that a two-day class closure, initiated the day after reaching a 10% absentee rate, could mitigate infection [50]. After a 2012 law was revised, the closure period for influenza was extended from “at least two days after the onset of fever” to “at least two days after the onset of fever and five days after the onset of illness” [52], which may have reduced in-class transmission opportunities. A comparative study found that this policy change was effective in preventing infection once absences exceeded 10%, although no reports discussed the optimal duration of class closures [55].

In this study, a longer duration of class closure reduced the number of infected students, indicating the effectiveness of closures. However, extended closures may increase the risk of negative impact beyond infectious diseases [20–27]. Further, class closures that minimize the number of absences occur when the number of infected students exceeds around 5.0%, with a closure duration of four days. Compared with previous studies, our value of 5% reflects a lower infection threshold. However, this result suggests that the total number of absences due to influenza (both infected students and those staying at home) could be smaller. Therefore, the duration of class closures should be examined in greater detail according to the purpose of the countermeasure. A few studies have assessed the effects of class closures on seasonal influenza; however, the study purpose and methods used varied [14,16]. Therefore, specific evaluation methods should be established [38]. No studies have examined efficiency.

Our mathematical model offers a framework to determine the optimal timing and duration of efficient class closures to prevent infections and minimize absenteeism. Our results inform discussions about absentee efficiency in schools. Countermeasures to deter school epidemics include adjusting school routines such as afterschool activities, school schedules, and small class sizes [56]. In addition, participation in special events, such as school activities, examinations, and sports matches, during an epidemic should be carefully considered, as they are important for students’ development. Appropriate evidence-based decisions can protect students from infectious diseases, while facilitating their educational activities. Our results provide insights into maintaining this balance.

Further, we indicated that schools could use collected data, such as student absences and community infection rates, to predict epidemics. While factors such as vaccination status [57] and household transmission risks [58,59] are beyond the control of school staff, using available data can still provide valuable insights. Our model was based on data available to schools. In Japan, school policymakers can use the Automatic Information Sharing System for School Absentees (a web-based system for reporting infections) to access information on infections in other schools and the Prefectural Surveillance Center of Infectious Disease to access information on infections in the community. By using these data, school administrators can make timely and appropriate decisions regarding class closures.

Class closures for seasonal influenza are typically reactive decisions made in response to in-class outbreaks. Limited evidence is available regarding guidelines on class closures during influenza outbreaks [60]. The class closure timing and duration identified in this study will help school administrators decide when to close classes (for example, to control the outbreak) or continue educational activities. By optimizing the efficiency of class closures, schools can minimize the spread of infection while reducing the burden of absenteeism, ultimately protecting both students’ health and education. Future studies should provide further scientific evidence to help schools make appropriate decisions.

This study has some limitations. First, we disregarded factors such as vaccination, family history, and personal behavior, which may have affected the prevalence of seasonal influenza in schools. Second, outside of class, students are at risk of influenza transmission in school clubs [61], gatherings among friends [62], and sports clubs [63]. In addition, attending classes at cram schools or preparatory schools and using public transport are also of risk. We just incorporated these risks in the simplest form in this study. Further studies are required to evaluate the influence of these risk settings on epidemics in detail. But for the timely prediction of seasonal influenza in schools, the prediction model should be simple and should use routinely collected data, similar to our model. We believe our results are useful and contribute to a more detailed examination of class closures.

## Conclusion

Reactive class closures for seasonal influenza were most efficient when the number of newly infected students exceeded around 5.0%, and the closure duration was four days. Although class closures had only a modest effect on reducing the number of infected students, they represent an effective strategy for controlling infection spread. The optimal class closure strategy balances long-term performance with potential variations. Our findings guide decision-making regarding efficient class closures to mitigate the spread of infection and minimize students’ absenteeism due to infectious diseases.

## Supporting information

S1 Fig Time series of the number of newly infected persons in high schools and the community. (PDF)

S1 Table Parameter estimation results. (DOCX)

S2 Table Reproduction number. (DOCX)
