# Supplementary figures and images for: Class-specific school closures for seasonal influenza: Optimizing timing and duration to prevent disease spread and minimize educational losses
Source: PLoS One. 2025 Jan 23;20(1):e0317017. doi: 10.1371/journal.pone.0317017 (PMC11756796; doi:10.1371/journal.pone.0317017)

2016 - 2017 season

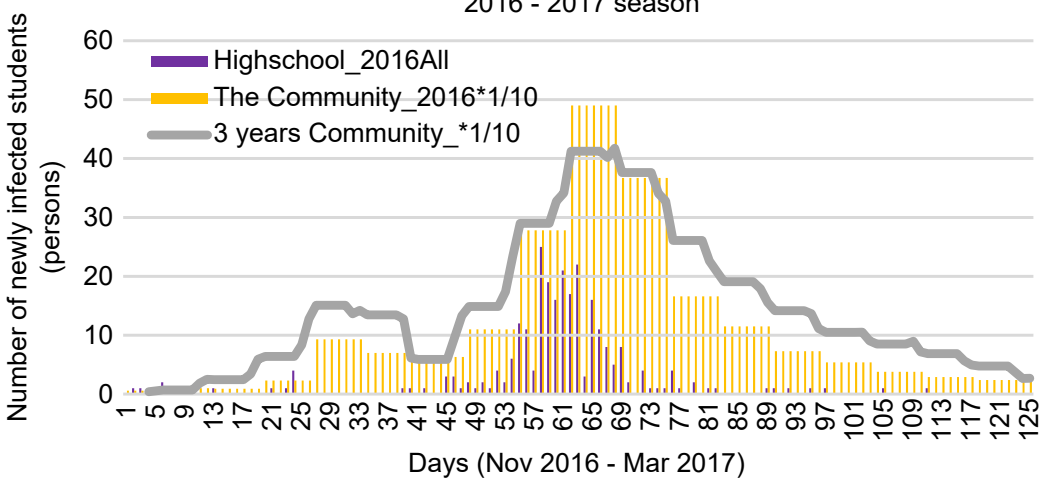

2017 - 2018 season

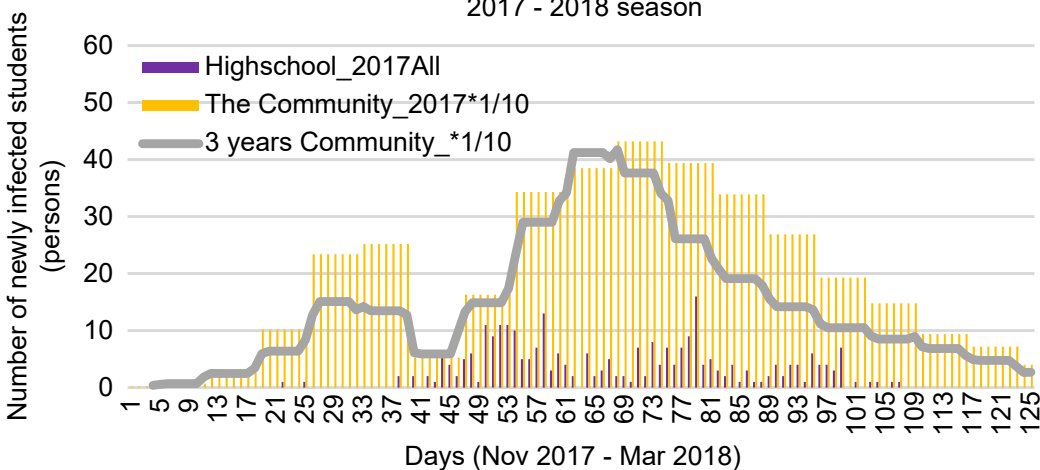

2018 - 2019 season

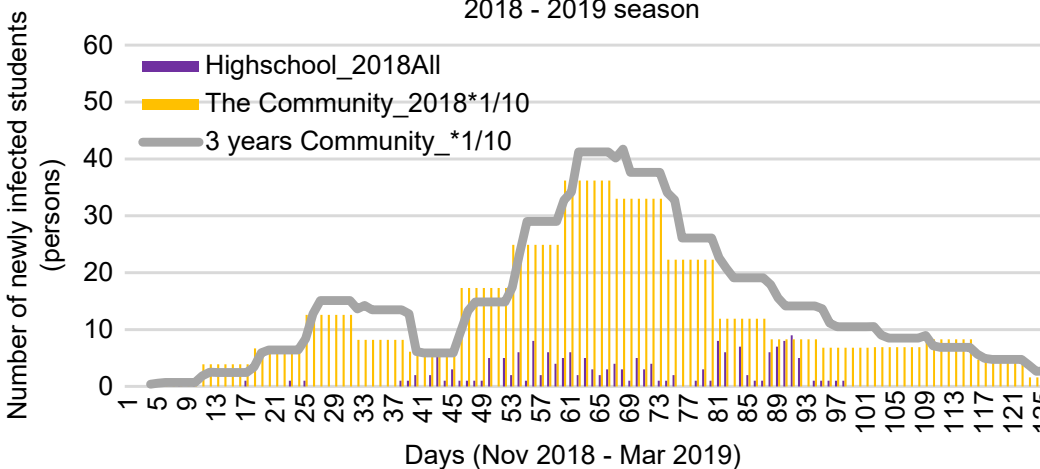

Supplement: S1 Fig — (PDF) [file pone.0317017.s001.pdf]
