# Supplementary material for: Class-specific school closures for seasonal influenza: Optimizing timing and duration to prevent disease spread and minimize educational losses
Source: PLoS One. 2025 Jan 23;20(1):e0317017. doi: 10.1371/journal.pone.0317017 (PMC11756796; doi:10.1371/journal.pone.0317017)
Supplement: S1 Table — (DOCX) [file pone.0317017.s002.docx]

**S1 Table. Parameter Estimation Results.** The parameters are within class (*β*_cl_), within grade (*β*_gr_), within school (*β*_sc_), between schools (*β*_all_), community (*β*_1_), intercept of community infections (*β*_0_), and time series of infection force (*s_1_* to *s_5_*). *S_3_* is fixed to avoid uncertain parameters.

|  | **2016–2017** | **(95% CI)** | **2017–2018** | **(95% CI)** | **2018–2019** | **(95% CI)** |
| --- | --- | --- | --- | --- | --- | --- |
| ***β_cl_*** | 0.011116 | (0.008115, 0.014117) | 0.003276 | (0.001889, 0.004662) | 0.013354 | (0.008802, 0.017907) |
| ***β_gr_*** | 0.000526 | (0.000000, 0.001054) | 0.000328 | (0.000036, 0.000619) | 0.000359 | (0.000000, 0.001305) |
| ***β_sc_*** | 0.000001 | (0.000000, 0.000221) | 0.000120 | (0.000000, 0.000278) | 0.000536 | (0.000064, 0.001009) |
| ***β_all_*** | 0.000255 | (0.000201, 0.000310) | 0.000208 | (0.000172, 0.000243) | 0.000183 | (0.000082, 0.000284) |
| ***β_0_*** | 0.000402 | (0.000201, 0.000602) | 0.000001 | (0.000000, 0.000115) | 0.000929 | (0.000495, 0.001364) |
| ***β_1_*** | 0.000002 | (0.000000, 0.000003) | 0.000001 | (0.000000, 0.000002) | 0.000004 | (0.000002, 0.000007) |
| ***s_1_*** | 0.235093 | (0.000000, 0.594636) | 0.102343 | (0.000000, 3.376192) | 0.117873 | (0.000000, 0.307828) |
| ***s_2_*** | 0.659716 | (0.410053, 0.909380) | 1.307057 | (0.887091, 1.727023) | 0.005266 | (0.000000, 0.169237) |
| ***s_4_*** | 0.000078 | (0.000000, 0.211465) | 1.148470 | (0.796523, 1.500418) | 0.002910 | (0.000000, 0.138117) |
| ***s_5_*** | 0.014122 | (0.000000, 0.477938) | 0.041591 | (0.000000, 1.633836) | 0.002792 | (0.000000, 0.193958) |
