# Supplementary material for: Class-specific school closures for seasonal influenza: Optimizing timing and duration to prevent disease spread and minimize educational losses
Source: PLoS One. 2025 Jan 23;20(1):e0317017. doi: 10.1371/journal.pone.0317017 (PMC11756796; doi:10.1371/journal.pone.0317017)
Supplement: S2 Table — (DOCX) [file pone.0317017.s003.docx]

**S2 Table. Reproduction Number.** *β* is the maximum likelihood estimate, and *R*(t) is the reproduction number calculated using B-spline.

*ss(*t) is the force of infection expressed as a B-spline (*S_cl_*(t) = 40, *S_gr_*(t) = 200, *S_sc_*(t) = 400, *S_all_*(t) = 1,600), and the reproduction number R(t) is *R*(t) = *ss*(t) (*β_cl_* × *S_cl_*(t) + *β_gr_* × *S_gr_*(t) + *β_sc_* × *S_sc_*(t) + *β_all_* × *S_all_*(t)) × 2.

|  |  | **Within class** | **Within grade** | **Within school** | **Between schools** |  |
| --- | --- | --- | --- | --- | --- | --- |
|  | **Parameter** | ***β_cl_*** | ***β_gr_*** | ***β_sc_*** | ***β_all_*** | **Total of R(t)** |
| **2016–2017** | β | 0.011116 | 0.000526 | 0.000001 | 0.000255 |  |
|  | Max of ss(t) | 0.72 | 0.72 | 0.72 | 0.72 |  |
|  | Peak of R(t) | 0.64 | 0.15 | 0.00 | 0.59 | 1.38 |
| **2017–2018** | β | 0.003276 | 0.000328 | 0.000120 | 0.000208 |  |
|  | Max of ss(t) | 1.10 | 1.10 | 1.10 | 1.10 |  |
|  | Peak of R(t) | 0.29 | 0.14 | 0.11 | 0.73 | 1.27 |
| **2018–2019** | β | 0.013354 | 0.000359 | 0.000536 | 0.000183 |  |
|  | Max of ss(t) | 0.50 | 0.50 | 0.50 | 0.50 |  |
|  | Peak of R(t) | 0.53 | 0.07 | 0.21 | 0.29 | 1.11 |
